# Atums Green Conjugated Polymer Heterojunction Films as Blue-Sensitive Photodiodes

**DOI:** 10.3390/polym17131770

**Published:** 2025-06-26

**Authors:** Zahida Batool, Razieh Firouzihaji, Mariia Babiichuk, Aria Khalili, John C. Garcia, Jau-Young Cho, Preeti Gahtori, Lukas Eylert, Karthik Shankar, Sergey I. Vagin, Julianne Gibbs, Alkiviathes Meldrum

**Affiliations:** 1 Department of Physics, University of Alberta, Edmonton, AB T6G 2E1, Canadafirouzih@ualberta.ca (R.F.);; 2 Institute of Physics, The Islamia University Bahawalpur, Bahawalpur 63100, Pakistan; 3 Nanotechnology Research Centre, National Research Council of Canada, Edmonton, AB T6G 2M9, Canada; akhalili@ualberta.ca (A.K.);; 4 The Department of Mechanical Engineering, University of Alberta, Edmonton, AB T6G 2E1, Canada; 5 Department of Electrical and Computer Engineering, University of Alberta, Edmonton, AB T6G 1H9, Canadakshankar@ualberta.ca (K.S.); 6 Department of Chemistry, University of Alberta, Edmonton, AB T6G 2G2, Canada; gahtori@ualberta.ca (P.G.); julianne.gibbs@ualberta.ca (J.G.); 7 Heinz Maier-Leibnitz Zentrum (MLZ), Technical University of Munich, Lichtenbergstraße 1, 85748 Garching bei München, Germanyvagin@tum.de (S.I.V.)

**Keywords:** asphaltene, conjugated polymer, Atums Green, light sensor, optical detection, fluorescence, blue light, bulk heterojunction

## Abstract

Conjugated polymers (CPs) offer many attractive features for photodiodes and photovoltaics, including solution processability, ease of scale-up, light weight, low cost, and mechanical flexibility. CPs have a wide range of energy gaps; thus, the choice of the specific polymer determines the optimum operational wavelength range. However, there are relatively few CPs with a strong absorption in the blue region of the spectrum where the human eye is most sensitive (440 to 470 nm) and none with an energy gap at 2.75 eV (450 nm), which corresponds to the peak of the CIE-1931 *z*(*λ*) color-matching function and the dominant blue light emission wavelength in computer and smartphone displays. Blue-light detectors in this wavelength range are important for light hazard control, sky polarization studies, and for blue-light information devices, where 450 nm corresponds to the principal emission of GaN-based light sources. We report on a new CP called Atums Green (AG), which shows promising characteristics as a blue-light photodetection polymer optimized for exactly this range of wavelengths centered around 450 nm. We built and measured a simple photodetector made from spin-coated films of AG and showed that its photosensitivity can be improved by the addition of asphaltene, a low-cost carbonaceous waste product.

## 1. Introduction

Light detectors with high sensitivity in the blue region of the spectrum are needed for light hazard control, solar UV monitoring, sky polarization, and for blue-light information and communications devices [1,2,3,4]. Silicon photodetectors exhibit reduced quantum efficiency in the blue and near-UV regions due to the shallow absorption depth for high-energy photons and significant surface recombination losses [5,6]. These limitations have spurred growing interest in developing blue-sensitive optical detectors for applications in fluorescence imaging, short-wavelength communication, and environmental sensing, in addition to the above-mentioned applications [7]. Emerging materials such as wide-bandgap semiconductors (e.g., GaN and ZnO) and low-dimensional nanomaterials (e.g., perovskites and quantum dots) are potential alternatives to silicon due to their absorption in the blue and UV wavelength range [8,9].

Organic materials are also widely investigated for wavelength-specialized sensors [10], but a comparatively small fraction of them are polymers. While inorganic devices tend to retain the highest photosensitivity (e.g., see recent work in Ref. [11]) polymer-based organic semiconductors offer the potential for thin, lightweight, flexible, solution-processable, and cost-effective devices for novel photosensors and photovoltaics [12]. Moreover, a wide range of fairly simple processing conditions allows much flexibility for performance improvements [13]. Conjugated polymers (CPs) are thus an attractive option for novel photosensors, but there are—to the best of our knowledge—none with a strong absorption band centered on the critical wavelengths around 450 nm. Atums Green (AG), a CP we recently developed and reported for the first time around two years ago [14], is just such a polymer. It has a strong absorption centered at ca. 450 nm, in the heart of the blue spectral region. Thus, AG seems potentially interesting for blue-light-sensitive photodiodes.

Despite these appealing features, polymer-based light-absorbing devices (and polymer photovoltaics) face several challenges owing to comparatively poor charge separation and modest charge extraction, which limit their achievable efficiency [15]. Blends of conjugated polymer (CP) donors with acceptor materials like fullerenes present a potential solution to the efficiency problem. This is because the interpenetrating network in bulk heterojunctions (BHJs) can separate photo-excited charges more effectively. The fullerene compound known as PCBM (i.e., [6,6]-phenyl-C_61_-butyric acid methyl ester) provides effective charge dissociation on picosecond time scales when blended into BHJs with an organic co-material, leading to vastly improved charge separation [16,17,18,19,20] but the fullerenes suffer performance limitations owing to their relatively weak absorption in the UV-visible region of the solar spectrum, modest carrier mobility, and various morphological issues [18]. Moreover, due to its laborious synthesis procedures, PCBM is quite expensive, with prices currently above USD 3000–4000 per gram from well-known chemical suppliers. For these reasons, semiconductor quantum dots (QDs) such as CdSe, CdTe and PbTe have been evaluated as alternative electron acceptors for hybrid organic solar cells that feature tunable absorption across the visible spectrum [21], but they frequently contain toxic and environmentally restricted elements [22]. Graphene quantum dots (GQDs) are believed to have lower toxicity [23] and have thus been investigated for various optoelectronic, energy storage and conversion, photovoltaic, and sensing applications (see for example, Ref. [24] and numerous citations therein). but they are also currently far too expensive to synthesize in quantities sufficient for large-scale device production.

An interesting alternative that has so far seen fairly little investigation is to use asphaltene in place of PCBM or GQDs for charge separation in organic polymer hybrids. Asphaltene is an abundant waste product associated with the production of heavy crude oil produced in the extraction of bitumen [25,26]. It is inexpensive and abundant, coming mainly as a waste product from hydrocarbon production, and it absorbs principally in the blue and UV parts of the spectrum. Asphaltene consists of a mixture of polycyclic aromatic hydrocarbons containing various heteroatoms including sulfur, nitrogen, and oxygen, a mix of trace metals, including vanadium and nickel, and nanoscale carbon particulates, including graphene quantum dots. Molecular dynamics simulations [27] as well as a few experimental reports [28] indicate that asphaltene can behave as a “non-fullerene acceptor” (NFA) in organic photodiodes, suggesting that it could perform as a low-cost additive for hybrid polymer light detectors and light energy harvesters. Given its abundance and similarities to certain additives used for polymer-based BHJ photodetectors, we wanted to determine whether asphaltene might be able to more directly serve as an alternative acceptor in an AG photodiode optimized for the important 450-nm wavelength range.

## 2. Materials and Methods

### 2.1. Materials

All chemicals and reagents were used as received unless otherwise indicated.

### 2.2. Preparation of Asphaltene-in-Chloroform (AC) Solutions

GQDs were prepared based on the following procedure in our previous report [29]. First, asphaltene as a precursor for A-Type GQDs was separated from Athabasca bitumen. To synthesize A-type GQDs, the asphaltene powder (100 mg) was dispersed in chloroform (5 mL) and ultrasonicated for three hours in an ultrasonic bath (VWR Ultrasonic Cleaner; Radnor, PA, USA). The as-synthesized AC-GQDs were separated by filtration using a syringe filter (PTFE, 0.2 µm).

### 2.3. Preparation of Atums Green (AG)

The AG polymer was prepared following established procedures [14]. The dibromo-monomer (100 mg, 0.122 mmol, 1 eq.) and diboronic acid monomer (91 mg, 0.122 mmol, 1 eq.) were dissolved in tetrahydrofuran (THF), degassed by three freeze–pump–thaw (FPT) cycles, and then clean, degassed K_3_PO_4_ (94 mg) was added to the solution. Next, Pd_2_(dba)_3_ (0.94 mg) and 2-(dicyclohexylphosphino)biphenyl (1.44 mg) were dissolved in 1 mL of degassed THF and added to the reaction mixture, which was then heated to 60 °C and stirred gently for one week. This resulted in the gradual formation of yellow precipitate which was then filtered off after cooling back to room temperature and then re-suspended in methanol and stirred for several hours. The precipitate was dried and then dissolved in 10 mL of dichloromethane and stirred with 1 M aqueous NaCN for several hours under ambient conditions. After washing with water, the precipitate was filtered off and dried to yield polymer with Mn ≈ 50 kDa and Đ ≈ 5. NMR, Raman spectroscopy, and gel permeation chromatography on the resulting material were all reported by us previously [14].

### 2.4. Preparation of AG—AC Mixtures and Thin Films

Initially, the as-prepared AG was dissolved in chloroform at a concentration of 10 mg/mL to form yellowish clear solutions. The asphaltene samples were dissolved in chloroform at the same concentration, producing a clear but dark yellowish-brown solution. These solutions were then mixed with additional chloroform to provide mixed solutions with AG-AC concentrations of 5-5, 5-3, 5-2, 5-1, 5-0.5, 5-0, and 0-5 (all values in mg/mL). To form thin films, the solutions were spin-coated on quartz or silicon wafers using a Ni-Lo 5 XL spin coater (Ottawa, ON, Canada). The wafers were first cleaned with Piranha solution in order to remove any organic contaminants. Approximately 300 μL of the respective solutions was applied with a spin-coating speed of 700 RPM on quartz wafers. The spin-coating time was 60 s, after which none of the solvent remained and the films appeared visually uniform and continuous. For electrical devices, the solutions were instead spin-coated on indium tin oxide (ITO) coated glass substrates which act as an active layer (Ossila S111 ITO glass substrates; Sheffield, South Yorkshire, UK). After spin coating, aluminum top contacts were subsequently deposited by electron beam evaporation at a pressure of 10^−7^ Torr and a deposition rate of 1.5 nm/min to produce Al films with a thickness of 150 nm.

### 2.5. Fluorescence Spectroscopy

The fluorescence of the solution and thin films was excited with the combined 352 and 364 nm lines of a UV-optimized Ar^+^ ion laser. The emitted fluorescence was collected with a 1-mm-diameter optical fiber, passed through a 400-nm longpass filter, and sent to wavelength-calibrated Ocean Optics (Orlando, FL, USA) miniature spectrometer. Intensity calibrations were performed using a reference blackbody lamp with a nominal color temperature of 2960 K. Absolute quantum efficiencies were measured in an integrating sphere with a 365 nm excitation source.

### 2.6. Time Resolved Fluorescence (TRF)

Time-resolved photoluminescence (TRPL) was measured using an Alphalas (Göttingen, Germany) Picopower 375-nm laser as the excitation source. This laser has nominally 30 ps pulses and it was adjusted to have a frequency of 5 MHz. The emission was collected with the same optical fiber, passed through the same 400 nm longpass filter, and sent instead to a Becker-Hickl (Berlin, Germany) HPM-100-40 single-photon-counting detector. The decay traces were registered with a Becker-Hickl SPC-130IN single photon counting module.

### 2.7. Absorption Spectroscopy

The absorption spectra of the thin films were measured using a fiber-coupled Thorlabs (Newton, NJ, USA) SLS204 deuterium tungsten lamp. The fiber was directed into a lensed wafer holder holder with a second fiber interfaced to the opposite side. All the absorbance spectra were measured relative to the corresponding uncoated wafer.

### 2.8. Thin Film Thickness

Thickness measurements were performed using an Alpha-step IQ profilometer (KLA Instruments, Milpitas, CA, USA) on thin films of pure polymer and its blend with ACC. The two-dimensional surface topography profile has sub-8-Å step height repeatability and sub-Å resolution. The thin films were prepared on fused quartz substrates by spin coating the pure polymer and blend solutions.

### 2.9. Spectroscopic Ellipsometry

In order to measure the optical constants, a set of thin films was prepared on silicon wafers as described above (see Section 2.4). Ellipsometric Ψ and Δ datasets were obtained on a JA Woollam (Lincoln, NB, USA) M-2000 ellipsometer over a range of incidence angles from 50 to 80 degrees, and the resulting data were modeled using the CompleteEase (v. 6.73) software package from Woollam. The optical constants were found by spline fitting the data with the film thickness constrained to be within 5% of the measured values.

### 2.10. Transient Absorption

Femtosecond transient absorption measurements were performed using a HARPIA-TA Ultrafast Transient Absorption Spectrometer (Light Conversion, Vilnius, Lithuania). The setup was powered by an amplified femtosecond ytterbium laser (Carbide, Light Conversion), with 80 W power, 2 mJ pulse energy, 333 fs pulse duration, a central wavelength of 1030 nm, and a 40 kHz repetition rate. The laser output was split into two parts: one part was directed to an optical parametric amplifier (OPA, ORPHEUS-F, Light Conversion) to generate the pump pulses, while the other part was used to produce a white-light supercontinuum probe beam by focusing the 1030 nm light into a CaF_2_ crystal. The samples were excited by 365 nm pump pulses with pulse energies of approximately 700 nJ. Transient absorption spectra were recorded in the wavelength range of 400–800 nm. Pump-induced absorption changes were measured in transmission mode using an Andor (Belfast, Northern Ireland) Kymera 193i spectrometer, with the time delay between the pump and probe pulses precisely controlled from a few fs to 8 ns.

### 2.11. Ultraviolet and X-Ray Photoelectron Spectroscopy

Ultraviolet photoelectron spectroscopy (UPS) measurements were performed on the AC films in order to determine the work function, using an Axis-Ultra instrument (Kratos, Manchester, UK) with a He lamp (21.21 eV) excitation source. The HOMO (highest occupied molecular orbital) level of AC was determined from XPS spectra measured with the same instrument.

### 2.12. Scanning Electron Microscopy

SEM was performed in secondary electron imaging mode using a Hitachi (Tokyo, Japan) S4800 cold field emission FESEM operated at an accelerating voltage of 5 keV. Samples were prepared by spin coating films on a silicon wafer, followed by the application of a 15 nm gold coating in order to reduce charging. Some samples were cleaved for cross-sectional imaging in order to confirm the film thickness measurements.

### 2.13. Atomic Force Microscopy

Film roughness and thickness measurements were conducted using a Dimension 3100 atomic force microscope (AFM; Veeco Instruments Inc., Plainview, NY, USA). AFM tips with a curvature radius of approximately 8 nm and a cantilever spring constant of 5 N/m were used. Film roughness was measured in tapping mode with an amplitude setpoint below 0.6 V and a scan rate of 0.4 Hz. Measurements were performed on a 10 × 10 µm^2^ scanning area and repeated three times. Data were processed using Gwyddion software (v. 2.68).

### 2.14. Electrical Measurements

The electrical characteristics of the fabricated devices were measured using Keithley 2400 source meter interfaced to a probe station. Contact was made by gently placing the probes directly onto the electrical contacts and carefully testing for a consistent, repeatable response. All the measurements were performed at room temperature and ambient conditions. The time, voltage, and current scan functions of the Keithley (Solon, OH, USA) were used and the data were collected with the Kickstart (v.2.11.3) software program from Keithley. For optical measurements, a controllable 405-nm LED was placed above the active part of the devices. A system of lenses was used to ensure a fairly uniform illumination area. The LED power was measured with a Coherent PM10 power meter (Saxonburg, PA, USA).

## 3. Results and Discussion

**Figure 1:**
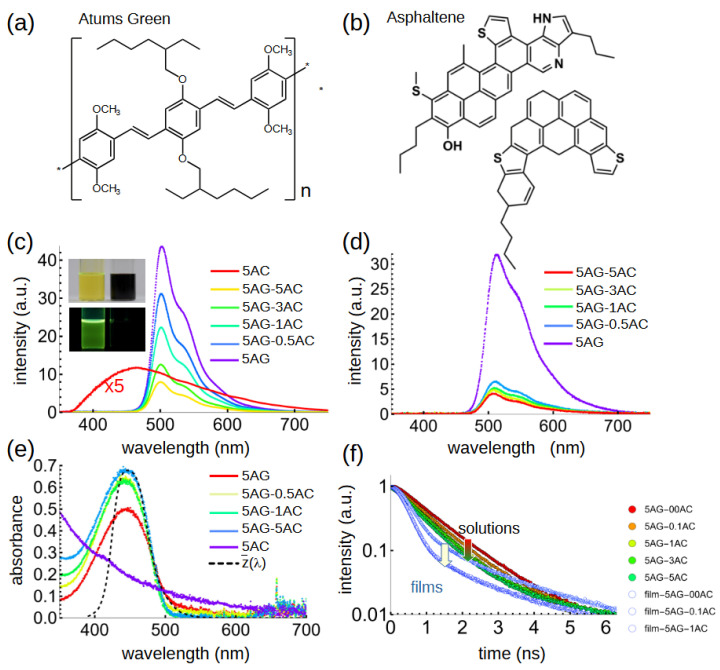
(**a**) Molecular diagram of AG. (**b**) Molecular illustration of AC. (**c**) Fluorescence of AG-AC solutions. The numbers refer to the concentration, in mg/mL of AG and asphaltene in chloroform. (**d**) Fluorescence of solid films spin-coated from AG-AC solutions. (**e**) Absorbance of AG-AC films. The dashed black line shows the CIE-1931 *z*(*λ*) color-matching function. (**f**) TRPL decays of AG-AC solutions with increasing AC content (red-to-green). TRPL decays of films are also shown (blue points).

The AG (Figure 1a) and AC (Figure 1b) solutions (both in chloroform) were luminescent but with different peak wavelengths and efficiencies (Figure 1c–f). AG has a bright green luminescence centered at ~500 nm and a quantum efficiency of 98% (thus, in our initial report of Atums Green we found it is a good laser gain medium [14]). In solution, it appears straw-yellow to the eye and has a well-defined absorption band at ca. 450 nm. In contrast, the fluorescence of AC peaked at 450 nm and had a quantum efficiency of only 4.6%, while the absorption showed a generally featureless increase with decreasing wavelength across the visible spectrum, becoming strong in the blue and UV region and giving the solution a brownish-orange appearance.

The fluorescence of the AG polymer solutions decreased monotonically with the addition of AC at various concentrations. No fluorescence from the AC could be observed in any of the mixed samples. At the same time, the mean fluorescence amplitude lifetime (fit with a weighted bi-exponential function) decreased from 0.94 to 0.75 ns with increasing AC concentration (Figure 1f), consistent with charge-separation-induced fluorescence quenching. The lifetimes are likely limited by the system response function of 0.4 ns; nevertheless, the clear, monotonic decrease is strong evidence in favor of the intended transfer of excited electrons from the polymer donor to the asphaltene acceptor moieties. This assertion is consistent with recent molecular dynamics simulations of a similar polymer–asphaltene system [27]. The absorbance peak shifted very slightly to ~448 nm with the addition of increasing concentrations of AC (Figure 1e).

In thin films produced by spin coating the above solutions, the behavior was similar but with some notable differences. The AG luminescence has a more extended long-wavelength tail, consistent with intra- and inter-chain interactions in many other conjugated polymers [30]. However, the quenching effect in mixed AG–AC films was much stronger than it was in solution; even at a low AC ratio of 0.1 mg/mL (compared to 5 mg/mL of AG in the original solutions), the fluorescence intensity decreased by ~90% and continued to slightly decrease even more with the addition of more AC into the films (Figure 1b). The absorbance peaked at ~445–450 nm for different AC concentrations, very close to that of the *z*(*λ*) tristimulus function which is shown as a dashed line in Figure 1c. The fluorescence lifetimes of the films were also even shorter, notably non-exponential, and strongly decreased with the addition of more AC (Figure 1d). The close proximity of the AC and AG moieties in solid films thus significantly enhanced the quenching effect, suggesting once again that AC has the intended charge-separating effect.

**Figure 2:**
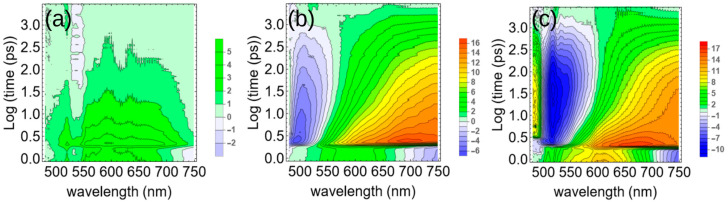
Transient absorption spectroscopic maps for (**a**) 5AC, (**b**) 5AG, and (**c**) 5AG–5AC solutions in chloroform. Note that the vertical axis is log(time) in picoseconds. The blue contours indicate ground-state bleaching and the red ones are excited-state absorption.

**Figure 3:**
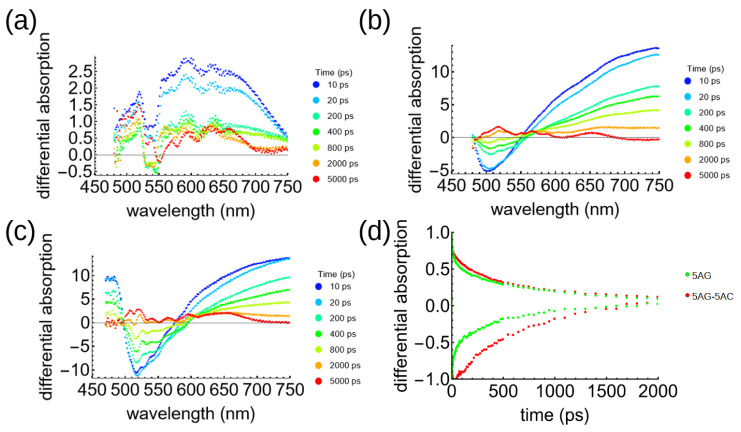
Transient absorption spectra for (**a**) 5AC, (**b**) 5AG, and (**c**) 5AG-5AC solutions in chloroform. The curves indicate later times from blue to red. (**d**) Transient absorption decays of the GSB in 5AG (green) and 5AG-5AC (red).

Transient absorption (TA) spectroscopy of the 0-5, 5-0, and 5-5 (AG-AC, mg/mL) solutions was performed in order to shed further light on the charge transfer dynamics. The AC solution showed excited state absorption (ESA) as a small positive differential signal across the visible spectrum; whereas, the AG showed a large negative signal across the absorption band in the range of 400-500 nm (Figure 2). This signal was present and even stronger in the 5-5 mixed sample, which is consistent with the greater absorbance of this sample as shown in Figure 1. These negative signals represent ground state bleaching (GSB), which can be reasonably inferred by the fact that the fluorescence of the 5-5 sample was nearly completely quenched. The GSB peak shifted from 500 nm in the polymer sample to 520 nm in the BHJ (Figure 3). The dynamics of these states are also considerably different, with the GSB decay significantly slower in the mixed sample, suggesting the presence of a long-lived charge-separated state [31]. Both the AG and AG–AC show pronounced ESA at longer wavelengths, but with similar decay timescales. This shows that the excited state is spectrally similar in the pure AG in its charged state—an observation that is generally consistent with other conjugated polymers that also have broad, featureless ESA spectra [32].

**Figure 4:**
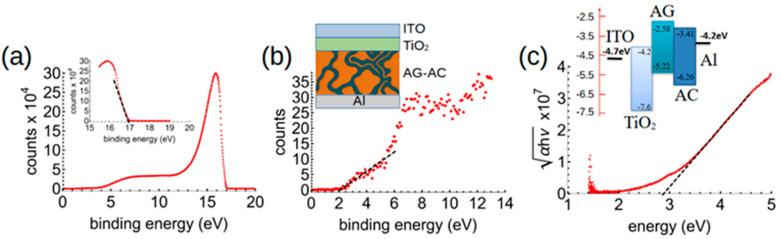
(**a**) UPS spectrum of AC coated films. (**b**) XPS spectrum of the same film, with a linear fit in the low-energy part. The film structure is illustrated diagrammatically in the inset. (**c**) Tauc plot with a linear fit from which the AC bandgap can be inferred. The resulting energy level diagram is shown in the inset, where the AG and AC energies are drawn separately but are in reality an interpenetrating bulk heterojunction.

The work function and HOMO–LUMO levels of AG were determined by us previously [14]. However, for AC it is unknown; therefore we performed XPS and UPS spectroscopy and a Tauc analysis on spin-coated pure AC films (Figure 4). The work function was obtained by subtracting the He I radiation energy of 21.21 eV from the measured high-binding energy cutoff at 16.95 eV. The XPS spectrum was used to estimate the position of the HOMO at 2.0 eV below the Fermi level (Figure 4), implying the HOMO is at –4.26 eV − 2.0 eV = –6.26 eV versus vacuum. The Tauc gap of AC was found to be ~2.85 eV, indicating that the LUMO energy lies close to –3.41 eV compared to vacuum. Accordingly, we obtained a work function of –4.26 eV and a HOMO energy of –6.26 eV for AC, relative to vacuum. These results, combined with those we already reported for AG [14], led to the energy level diagram shown in Figure 4c and further confirm that the AG–AC forms a donor–acceptor pair.

**Figure 5:**
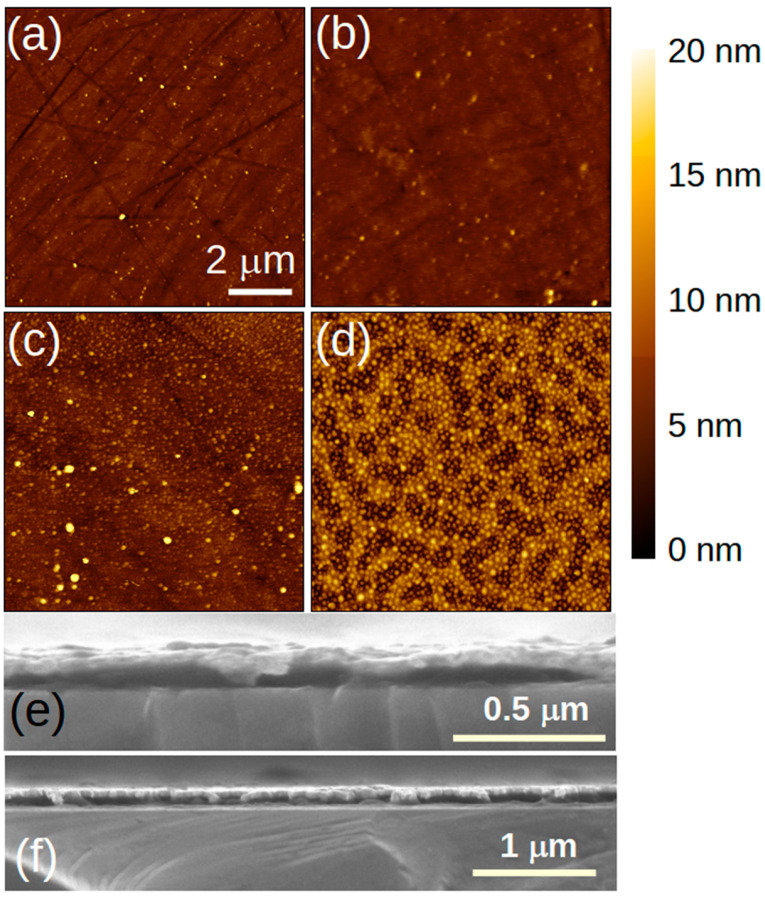
(**a**–**d**) shows AFM images of film spin coated from 5AG, 5AG–0.5AC, 5AG–1AC, and 5AG–5AC solutions. (**e**) Cross-sectional SEM image of an AG film. (**f**) Cross-sectional SEM image of a 5AG-5AC film. All the films were spin-coated onto silicon.

The physical structure of the films was determined by AFM and SEM. The spin-coated AG polymer films were quite smooth, with a root mean square roughness of 0.8 nm. The addition of AC caused an increasing roughness until it reached 2.3 nm in the 5-5 film (Figure 5). The AC appears as small clusters or islands with a mean lateral size of ~100 nm and a height of ~15 nm. One can see hints of a percolating network in the film topography (Figure 5d), suggesting that the AC has a tendency to coagulate within the thin films. This behavior has obvious implications for the applications of the AG–AC hybrid as a photosensing device. Cross-sectional SEM and contact profilometry showed that the AG films were ~48 nm thick. The films became thicker with the addition of AC, up to ~92 nm in the sample with the largest amount of AC added (5 mg/mL).

**Figure 6:**
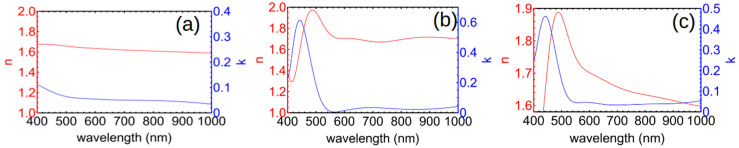
Optical constants obtained from ellipsometry for (**a**) 5AC, (**b**) 5AG, and (**c**) 5AG–5AC films spin coated on glass substrates.

Ellipsometry was performed on films made from pure AC, pure AG, and an equal mixture (5-5 films). Tauc–Lorentz and spline fitting of the raw ellipsometric *Ψ* and *Δ* values were used to obtain the optical constants, with the film thickness limited to within 10% of those obtained by SEM and profilometry in order to account for film thickness variations across the spin-coated samples. For films coated from pure AC, we find that the refractive index increased monotonically from 1.59 to nearly 1.68 on decreasing the wavelength from 1000 to 400 nm (Figure 6a). The imaginary part of the refractive index also increased with decreasing wavelength, consistent with the measured absorbance data. Pure AG films have a fairly high refractive index (around 1.7), increasing up to 1.9 at a wavelength of 490 nm (Figure 6b). The absorbance was close to but not exactly zero over most of the spectrum, possibly because of scattering or uncertainties in the fitting. The imaginary part of the refractive index showed a strong peak at ~445 nm, which is very close to the measured absorbance peak at 448–450 nm. In mixed films, we found that the optical constants act mainly like an effective medium or “blend” of the pure films (Figure 6c). With these results, >80% of the incident light incident on the 5AG–5AC film is absorbed, accounting for reflection from the back contact.

**Figure 7:**
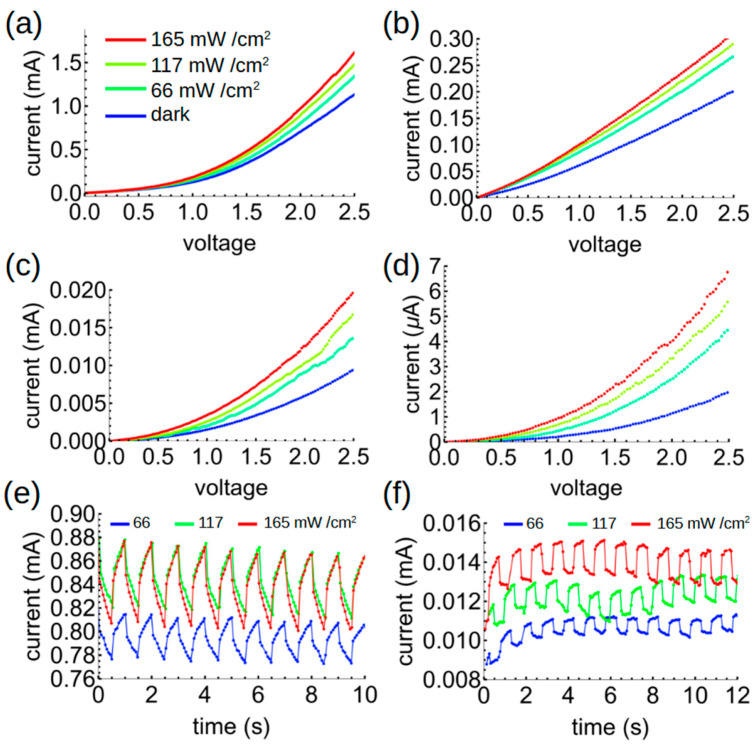
Current-voltage curves for (**a**) 5AG, (**b**) 5AG–0.5AC, (**c**) 5AG–1AC, and (**d**) 5AG–5AC devices. Photocurrent response as a function of time for (**e**) 5AG and (**f**) 5AG–1AC. Data are shown after a few seconds of stabilization.

**Figure 8:**
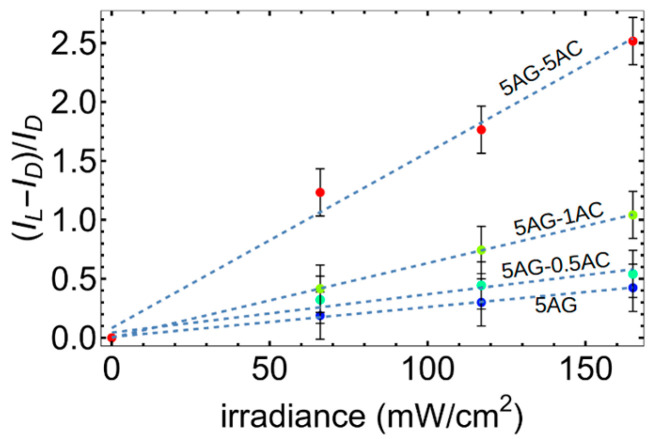
Photosensitivity as a function of irradiance for the pure AG and the three blends. The slope is steepest for the mixed 5AG–5AC film, indicating better charge separation and resulting photocurrents due to the presence of AC.

We next investigated the electrical properties of the hybrid polymer–asphaltene films under forward bias (with the positive terminal attached to the Al electrode and the negative terminal to the ITO). (Reverse bias—the more usual case for organic photodiodes—will be discussed below.) The data in Figure 7 illustrate several key results. First, we find an induced photo-current in all the samples which became easily observable at biases above 0.5 V. This photo-current increases with increasing irradiance. Second, the current decreases monotonically with increasing AC concentration in the films. This suggests that the current is limited by percolation networks, i.e., some of the charges get “stuck” in the hybrid network, reducing the measured current. However, the photocurrent—that is, the difference between the dark current and the current measured under illumination—increases with increasing AC concentration for all irradiances tested, indicating that charge separation between the AG and AC significantly enhances the photocurrent response in comparison to the dark current. This is clearly apparent in Figure 8, which shows that the photosensitivity, defined as the photocurrent over dark current given by *P_S_* = *(I_L_ − I_D_)/I_D_*, where *I_L_* and *I_D_* represent the light and dark currents, increases by an approximately order of magnitude in the 5-5 hybrid as compared to the pure AG films (under a 2.5 V bias) for all tested irradiances. The optimum responsivity, external quantum efficiency (EQE), and specific detectivity were found to be 0.22 A/W, 69%, and 1.4 × 10^9^ Jones in the pure AG films. These values decreased to 0.0085 A/W, 2.6%, and 4.2 × 10^8^ Jones in the 5-5 mixture. In all samples, the EQE is further limited by the high index of refraction of the ITO electrode compared to glass, which leads to reflection loss; however, the good index match between AG and ITO (close to 2 in the blue part of the spectrum, as in published databases) should minimize reflection at that boundary. Thus, we conclude that while charge separation in the AG–AC mixture strongly enhances *P_S_* due to efficient charge separation, it decreases the responsivity and the EQE due to poorer current extraction, likely because of incomplete percolating pathways. The response of the BHJs to an oscillating light source was, moreover, faster than that of the pure AG film (Figure 7e,f). With increasing AC concentration up to the sample 5-5, the response to an alternating light source becomes flat-topped, in comparison to pure AG films, which showed a slower rise and decay. While we could not determine the ultimate frequency limit of the material due to the limitations of our testing system, we can conclude that AC benefits the inherent device speed in addition to improving the photosensitivity.

We recall that AG and its blends with AC have an absorption peak at 450 nm, which is ideal for blue-light sensing and imaging applications, especially because AG matches closely with the blue CIE-1931 *z*(*λ*) spectrum and with the blue emission from computer monitors and smartphone screens. Thus, we checked to see if the extremely simple devices we built could detect blue light from a smartphone. We tested two models—an iPhone 9 and a OnePlus 7 Pro. The former peaks at 458 nm and the latter at 456 nm when the screen is illuminated in pure blue, as expected, very close to the absorption maximum of AG and AG–AC BHJs. While our devices are very simple one-layer BHJ photodiodes, they were, at times, barely able to measure blue light from smartphones placed a few centimeters away. While the results are promising for AG-based photodiodes, this result clearly shows that there is considerable room for improvement in the device responsivity (likely relating to percolation in the AC network, as suggested by the AFM results) as the key metrics are inferior to those for other photodetectors, including recent inorganic metal-insulator-semiconductor devices with good response at 450 nm [11].

**Figure 9:**
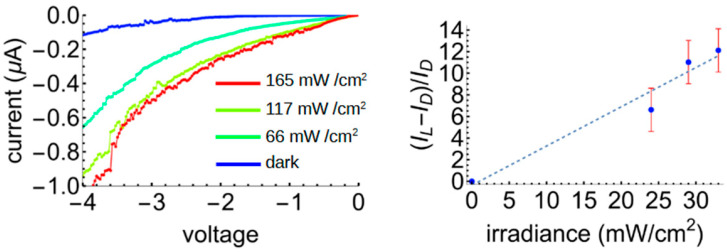
Right: IV curves for the 5AG–5AC devices under reverse bias. Left: the photoresponse as a function of the incident irradiance.

Many photodiodes operate under reverse bias due to the lower noise floor in that current direction. Therefore, we also measured the photoresponse of the 5-5 blend with the opposite polarity (Figure 9). The photosensitivity, defined as before, is indeed significantly larger in reverse for this sample (12.1 vs. 2.5 in forward bias) but the current is also much lower, reducing the responsivity to 0.9 mA/W and the EQE to 0.28%. Thus, even in reverse bias we have the same trade-off between the relative magnitude of the response (the photosensitivity) compared to the responsivity and external quantum efficiency.

**Figure 10:**
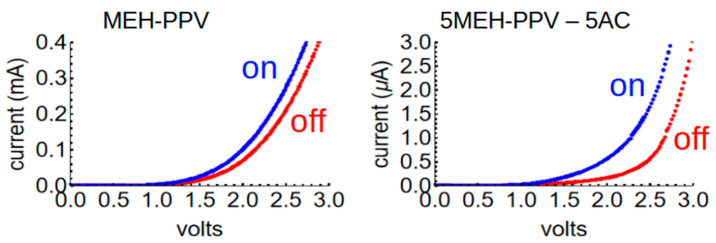
IV curves for an MEH–PPV device (left) and one made from a 5-5 mg/mL blend of MEH-PPV and AC (right).

Finally, we wanted to see if asphaltene provides the same properties in other CP films as it does in AG. To investigate this, another 5-5 sample was made, replacing AG with MEH-PPV (40 kDa/mol), a well-known CP which absorbs in the green (~530 nm peak) and is thus mimics, at least to some degree, the green CIE-1931 tristimulus function. The procedures followed to produce the photodiode were otherwise identical to those with AG. The results were strikingly similar (Figure 10). Whereas pure MEH–PPV has a high photocurrent and a high dark current, the 5-5 blend has much lower currents but a better photocurrent (i.e., the difference between the current under dark and illumination conditions). Thus, we believe that asphaltene has the potential to enhance charge separation and improve performance in polymer-based photodiodes in general. Further optimization will be desirable, however, especially relating to improving charge transport.

## 4. Conclusions

AG is a new green-fluorescent polymer reported for the first time two years ago [14]. It has an absorbance band that matches well with the blue-perceptive color region, and its absorption peak aligns with the GaN-based blue emission wavelength from computer monitors and cellphone screens. Materials with such idealized absorption characteristics are of significant interest for blue-light sensors, computer vision, and high-throughput color imagers. In this work, we built and tested simple BHJ photodiodes based on AG in the pure form and blended with asphaltene (AC). AC was chosen as the acceptor material because there are recent reports suggesting that asphaltene can assist in effectively separating charges in bulk heterojunction photodiodes, and it is thus a potential acceptor in conjugated polymer BHJs.

The results show that AG is indeed an effective material for blue-light-specialized photodiodes. When blended with AC, the photoresponse, defined as the photocurrent divided by the dark current, increases by an order of magnitude due to more effective charge separation than can be obtained in pure polymer films. The properties of AG–AC blends were investigated by fluorescence and absorption spectroscopy, ultrafast transient absorption, ellipsometry, XPS, UPS, SEM, and AFM. These complementary techniques provide a picture of the film morphology and optical properties, especially as they relate to the measured photodiode characteristics. Steady-state and time-resolved spectroscopic data confirm that AC enhances exciton dissociation and suppresses radiative recombination, supporting the observed improvement in the photoresponse. Ellipsometry and AFM reveal systematic changes in thickness and surface roughness as a function of AC content, which correlate with variations in film uniformity and percolation network formation. XPS and UPS analyses provided evidence of the energy level alignment and interfacial electronic structure, validating that the HOMO and LUMO offsets between AG and AC are sufficient for efficient charge transfer. The HOMO and LUMO levels for AC were found to be –6.26 and –3.41 eV, respectively, while those for AG are –5.22 and –2.58 eV. The films ranged from 48 to 92 nm thick, depending on the AC content, and the roughness ranged from 0.8 to 2.3 nm (RMS) with increasing asphaltene. These thicknesses are sufficient to account for >80% absorption at 450 nm for a double traverse of the mixed AG–AC films.

Together, these findings demonstrate how Atums Green can function as a novel, blue-optimized polymer photodiode and how asphaltene can act as a low-cost acceptor material that strongly contributes to enhanced charge separation. Although the present devices had a good photoresponse, they were limited in terms of responsivity, detectivity, and EQE due to comparatively poor charge transport in BHJ films. Nevertheless, the results point to clear strategies for later improvement. We anticipate that future work has considerable room to optimize the device architecture, combining the excellent photosensitivity and charge separation demonstrated here with improved charge collection, transport, and amplification methods. While asphaltene clearly offers promise as an economical acceptor material in organic BHJ devices, improvement in uniformity and quality will be necessary for it to achieve PCBM-like efficiencies.

## Data Availability

Data may be made available upon request.
